# Implications of Shortened Quarantine Among Household Contacts of Index Patients with Confirmed SARS-CoV-2 Infection — Tennessee and Wisconsin, April–September 2020

**DOI:** 10.15585/mmwr.mm695152a1

**Published:** 2021-01-01

**Authors:** Melissa A. Rolfes, Carlos G. Grijalva, Yuwei Zhu, Huong Q. McLean, Kayla E. Hanson, Edward A. Belongia, Natasha B. Halasa, Ahra Kim, Jennifer Meece, Carrie Reed, H. Keipp Talbot, Alicia M. Fry

**Affiliations:** ^1^CDC COVID-19 Response Team; ^2^Vanderbilt University Medical Center, Nashville, Tennessee; ^3^Marshfield Clinic Research Institute, Marshfield, Wisconsin.

To prevent further transmission of SARS-CoV-2, the virus that causes coronavirus disease 2019 (COVID-19), CDC currently recommends that persons who have been in close contact with someone with SARS-CoV-2 infection should quarantine (stay away from other persons) for 14 days after the last known contact.* However, quarantine might be difficult to maintain for a prolonged period. A shorter quarantine might improve compliance, and CDC recommends two options to reduce the duration of quarantine for close contacts without symptoms, based on local circumstances and availability of testing: 1) quarantine can end on day 10 without a test or 2) quarantine can end on day 7 after receiving a negative test result.^†^ However, shorter quarantine might permit ongoing disease transmission from persons who develop symptoms or become infectious near the end of the recommended 14-day period. Interim data from an ongoing study of household transmission of SARS-CoV-2 were analyzed to understand the proportion of household contacts that had detectable virus after a shortened quarantine period. Persons who were household contacts of index patients completed a daily symptom diary and self-collected respiratory specimens for 14 days. Specimens were tested for SARS-CoV-2 using reverse transcription–polymerase chain reaction (RT-PCR). Among 185 household contacts enrolled, 109 (59%) had detectable SARS-CoV-2 at any time; 76% (83/109) of test results were positive within 7 days, and 86% (94 of 109) were positive within 10 days after the index patient’s illness onset date. Among household contacts who received negative SARS-CoV-2 test results and were asymptomatic through day 7, there was an 81% chance (95% confidence interval [CI] = 67%–90%) of remaining asymptomatic and receiving negative RT-PCR test results through day 14; this increased to 93% (95% CI = 78%–98%) for household members who were asymptomatic with negative RT-PCR test results through day 10. Although SARS-CoV-2 quarantine periods shorter than 14 days might be easier to adhere to, there is a potential for onward transmission from household contacts released before day 14.

A CDC-supported study of household transmission of SARS-CoV-2 is currently ongoing in Nashville, Tennessee, and Marshfield, Wisconsin (*1*). Household contacts of an index patient who had symptoms compatible with COVID-19 for <7 days and laboratory-confirmed SARS-CoV-2 infection were eligible for the study if they had not had symptoms of an acute respiratory illness up to the date of the index patient’s illness onset.^§^ Enrolled household contacts were instructed to self-collect respiratory specimens (nasal swab only or nasal swab and saliva) and complete a daily symptom diary for 14 days.^¶^ The study protocol was reviewed and approved by the Vanderbilt University Medical Center’s and Marshfield Clinic Research Institute’s Institutional Review Boards and was conducted consistent with applicable federal law and CDC policy.**

For each household contact, the number of days from the index patient’s illness onset to 1) the day of first positive test result, 2) the day of first symptoms, or 3) the end of the follow-up period, whichever occurred first, was calculated. Survival analysis, accounting for left-censoring,^††^ was used to estimate the conditional probability that a household contact who was asymptomatic and whose specimens tested negative for SARS-CoV-2 by RT-PCR through day 5, 7, or 10 would remain asymptomatic and negative through day 14. Sensitivity analyses were conducted, excluding households with possible co-primary patients (households with household contacts who had illness onset or positive test <2 days after the index patient’s illness onset) and household contacts with possible tertiary transmission (household contacts who had a positive test >2 days after another nonindex household contact had a positive test result).

During April–September 2020, among 105 index patients, 185 household contacts were enrolled (median of one household contact per index patient, interquartile range [IQR] = 1–2; 45% of household contacts were male; median age of household contacts = 27 years, IQR = 15–45 years). Enrollment occurred a median of 4 days (IQR = 2–4 days) after the index patient’s illness onset and study follow-up concluded a median of 16 days (IQR = 15–17 days) after the index patient’s illness onset. Overall, 109 (59%) household contacts had SARS-CoV-2 detected in respiratory specimens during the follow-up period, with the first positive specimen collected a median of 5 days (IQR = 3–7 days) after the index patient’s illness onset. Among all infected household contacts, 76% (83 of 109) had infection detected within 7 days after the index patient’s illness onset and 86% (94 of 109) within 10 days.

The probability that a household contact who was asymptomatic and had negative RT-PCR test results through day 7 would remain asymptomatic with negative RT-PCR test results through 14 days after the index patient’s illness onset was 81% (95% CI = 67%–90%); the probability increased to 93% (95% CI = 78%–98%) if the household contact remained asymptomatic with negative test results through day 10 (Table). After excluding 22 households (including 45 household contacts) with possible co-primary index patients and 10 infected household contacts with possible tertiary transmission, the conditional probability that the contact would remain asymptomatic with negative RT-PCR test results through day 14 was 95% (95% CI = 81%–99%) if the person was asymptomatic and negative through day 10.

**TABLE:** Cumulative frequency and conditional probability of SARS-CoV-2 detection or symptoms over time among household contacts who received positive SARS-CoV-2 test results or developed symptoms — Tennessee and Wisconsin, April–September 2020

Main analysis	No. of days from index patient's illness onset	No. of household contacts (% of total)			Conditional probability of remaining asymptomatic with negative test results until day 14 % (95% CI)*
		SARS-CoV-2 detected	Symptomatic	Symptomatic or SARS-CoV-2 detected	
		n = 109	n = 122	n = 145	
All households and all household contacts included^†^	5	68 (62)	101 (83)	119 (82)	71 (57–81)
	7	83 (76)	110 (90)	130 (90)	81 (67–90)
	10	94 (86)	116 (95)	138 (95)	93 (78–98)
	14	104 (95)	121 (99)	141 (97)	—
**Sensitivity analyses**					
Excluding households with possible co-primary patients^§^	**—**	**n = 75**	**n = 84**	**n = 103**	**—**
	5	41 (55)	68 (81)	80 (78)	74 (59–84)
	7	52 (69)	74 (88)	88 (85)	80 (66–89)
	10	62 (83)	79 (94)	96 (93)	93 (77–98)
	14	70 (93)	83 (99)	99 (96)	—
Excluding household contacts that are possibly tertiary transmissions^¶^	**—**	**n = 99**	**n = 118**	**n = 135**	**—**
	5	68 (69)	99 (84)	117 (87)	76 (61–86)
	7	80 (81)	108 (92)	127 (94)	87 (72–94)
	10	88 (89)	113 (96)	127 (94)	95 (82–99)
	14	97 (98)	118 (100)	134 (99)	—
Excluding households with possible co-primary patients and household contacts that are possibly tertiary transmissions**	**—**	**n = 66**	**n = 81**	**n = 94**	**—**
	5	41 (62)	67 (83)	79 (84)	80 (64–89)
	7	50 (76)	73 (90)	86 (91)	86 (71–94)
	10	57 (86)	77 (95)	86 (91)	95 (81–99)
	14	64 (97)	81 (100)	93 (99)	—

* The conditional probability is the probability of remaining negative by reverse transcription–polymerase chain reaction and asymptomatic to day 14 after the index patient’s illness onset, given that the household contact has been negative and asymptomatic through day 5, 7, or 10. 95% CIs were estimated using Greenwood’s exponential CIs (Major Greenwood, Jr. [1926]. The Natural Duration of Cancer. Reports of Public Health and Related Subjects, Vol. 33, HMSO, London).

^†^ Analysis included 104 households and 185 household contacts.

^§^ Analysis included 82 households and 141 household contacts. Households were excluded if any household contact had illness onset or positive test <2 days after the index patient’s illness onset.

^¶^ Analysis included 104 households and 175 household contacts. Household contacts were excluded if they had a positive test >2 days after another nonindex household contact became positive.

** Analysis included 82 households and 132 household contacts.

## Discussion

Quarantine can stop onward transmission of SARS-CoV-2; however, adherence to a 14-day quarantine can be challenging. Analysis of data from an ongoing study of SARS-CoV-2 detection after exposure to an infected household member found an 81% chance that a household contact who had negative SARS-CoV-2 RT-PCR test results and was asymptomatic for 7 days after the index patient’s illness onset date would remain asymptomatic and continue to receive negative RT-PCR test results through 14 days. Conversely, one in five household contacts would become symptomatic or receive positive SARS-CoV-2 RT-PCR test results between day 7 and 14, suggesting that, compared with no quarantine, reducing quarantine to <14 days might decrease but not eliminate the risk for spreading SARS-CoV-2.

With consistent adherence, quarantine prevents transmission from persons who were exposed to the virus and who might become infectious, but who do not have symptoms or signs of infection (i.e., who are presymptomatic or who will remain asymptomatic). The length of quarantine is typically based on the known incubation period, or the interval between exposure to an infectious pathogen and the development of symptoms or signs of infection, which for SARS-CoV-2 ranges from 2 to 14 days.^§§^ However, quarantine efforts will not effectively reduce transmission if adherence is low. Evidence suggests that adherence to recommended quarantine during the COVID-19 pandemic varies and might be low in some settings (*2*,*3*). France, Belgium, and now some jurisdictions in the United States have shortened the quarantine period for persons exposed to someone with COVID-19 from 14 days to 10 or 7 days, but there is ongoing concern that shortening quarantine for all exposed persons could increase community transmission (*4*). Modeling studies suggest that combining a shorter quarantine with a timely diagnostic test at the end, to detect asymptomatic or presymptomatic infections, might carry some residual risk for transmission but could be an alternative to a 14-day quarantine period if the shorter quarantine length enhances compliance.^¶¶^^,^***

In this analysis of SARS-CoV-2 detection following household exposure, the more time that had passed since the index patient’s illness onset, the higher the likelihood that an asymptomatic household contact with negative SARS-CoV-2 test results would remain asymptomatic and RT-PCR negative. If the household contact remained asymptomatic with negative SARS-CoV-2 RT-PCR test results through day 7, the probability of their becoming symptomatic or having a positive RT-PCR test result the following week was 19%. However, this probability declined to 7% if the contact remained asymptomatic with negative RT-PCR test results through day 10. To minimize the potential risk for further spread, persons who have been released from a shortened quarantine should continue to monitor their health for symptoms of COVID-19, avoid close contact with others (including persons in their household), and cover their nose and mouth with a mask when around others for the remainder of the 14 days.^†††^

The findings in this report are subject to at least five limitations. First, the index patient’s illness onset date was used as a proxy for last exposure. This might not have been the actual date of last exposure, affecting calculations on timing of positive specimens and symptom onset. Second, chains of transmission are challenging to recreate with observational studies; however, the main findings were robust in several sensitivity analyses designed to account for possible misclassification of secondary infections. Third, household contacts were assumed to have acquired infection from the index patient; however, the possibility that some infections might have been introduced from the community cannot be excluded. Fourth, a highly sensitive assay was used to detect SARS-CoV-2 nucleic acids; in some settings, however, this type of testing might not be available or yield timely results. Finally, these findings might not be directly translatable to use of point-of-care assays, which yield more rapid results but with lower sensitivity.

A 14-day quarantine of all close contacts who are exposed to a person with COVID-19, such as in the household, is the most effective strategy to reduce the spread of COVID-19. Although persons might be more adherent to a shorter quarantine period, such a policy is not without risk for further spread. Timely access to a sufficiently sensitive test at the end of a shorter quarantine period will help identify household contacts with SARS-CoV-2 infection and might enable an effective shorter quarantine period for household contacts who remain asymptomatic and have negative test results, who pose lower risk for further spread of COVID-19.

**Summary**

**What is already known about this topic?**

After exposure to COVID-19, a 14-day quarantine period can prevent further spread but might be challenging to maintain.

**What is added by this report?**

Among persons exposed to COVID-19 in the household who were asymptomatic and had negative laboratory test results through 7 days after symptom onset in the index patient, 19% experienced symptoms or received positive test results in the following week.

**What are the implications for public health practice?**

A shorter quarantine after household exposure to COVID-19 might be easier to adhere to but poses some risk for onward transmission. Persons released from quarantine before 14 days should continue to avoid close contact and wear masks when around others until 14 days after their last exposure.

## Footnotes

*
https://www.cdc.gov/coronavirus/2019-ncov/if-you-are-sick/quarantine.html .

†
https://www.cdc.gov/coronavirus/2019-ncov/more/scientific-brief-options-to-reduce-quarantine.html .

§ Index patients (or their parent/guardian) were asked “Have all other members of their household had fever, cough, cold, or other respiratory symptoms?” [Marshfield, Wisconsin site] or “Has anyone in your home, besides you, been sick with a fever, cough, cold, or flu-like symptoms in the 7 days before your illness began?” [Nashville, Tennessee site]. If the response was yes, the household was ineligible to participate.

¶ The following signs and symptoms were solicited from participants on the daily diary: fever/feverishness, cough, sore throat, runny nose, trouble breathing or shortness of breath, nasal congestion, chills, fatigue or feeling run down, wheezing, chest tightness or chest pain, abdominal pain, diarrhea, vomiting, headache, muscle or body aches, and loss of taste or smell.

** 45 C.F.R. part 46.102(l)(2), 21 C.F.R. part 56; 42 U.S.C. Sect. 241(d); 5 U.S.C. Sect. 552a; 44 U.S.C. Sect. 3501 et seq.

†† Household contacts who had positive SARS-CoV-2 RT-PCR test results on the date of enrollment were considered left-censored because the date of initial infection was not observed during the study period.

§§
https://www.cdc.gov/coronavirus/2019-ncov/hcp/faq.html#Transmission .

¶¶
https://www.medrxiv.org/content/10.1101/2020.10.27.20211631v1.full.pdf .

*** https://www.medrxiv.org/content/10.1101/2020.08.21.20177808v3.full.pdf.
